# Residual Feed Intake as a Behavioral, Nutritional and Economic Criterion in Poultry Production

**DOI:** 10.3390/ani15081115

**Published:** 2025-04-12

**Authors:** Anastasiya Ramankevich, Sofiia Danko, Rafał Banaszkiewicz, Kornel Kasperek, Grzegorz Zięba

**Affiliations:** Institute of Biological Bases of Animal Production, Faculty of Animal Science and Bioeconomy, University of Life Sciences in Lublin, 13 Akademicka Str., 20-950 Lublin, Poland; anastasiya.ramankevich@up.lublin.pl (A.R.); grzegorz.zieba@up.lublin.pl (G.Z.)

**Keywords:** breeding, feed efficiency, feed intake, egg production

## Abstract

Rising feed costs in the poultry industry have the greatest impact on the price of the final product. To address this issue, several indicators have been developed to assess feed efficiency. One such indicator is residual feed intake (RFI) which represents the difference between actual and expected feed consumption. A lower RFI indicates better performance, making it a valuable trait for sustainable poultry production. RFI is influenced by several factors, including metabolism, body temperature regulation, arrangement of feathers, behavior and physical activity, all of which affect energy expenditure. The trait has moderate heritability, enabling genetic improvement through selective breeding. Accurate calculation of RFI is essential for effective selection, taking into account growth and maintenance needs. Understanding these factors enables breeders to increase feed efficiency, reduce production costs and promote sustainability within the industry.

## Simple Summary

The growing human population is associated with an increased demand for food including poultry industry products. In this industry, the majority of costs affecting the final price arise from feed expenses. Therefore, efficient feed utilization in poultry farming is important to reduce costs and protect the environment. Residual feed intake (RFI) is a metric used to assess which birds consume less feed than expected based on their needs while maintaining optimal production and health indicators. Scientists and breeders aim to gain a deeper understanding of the physiological and genomic basis of birds in terms of efficient feed utilization, namely what mechanisms determine the efficiency of nutrient digestion and absorption. Since this trait is partially heritable, it can be enhanced through the careful selection of breeding animals. However, both selection processes and the calculation of RFI must be conducted with precision, taking into account the production rates of birds due to the possible negative impact on them. Improved feed utilization leads to reduced resource consumption, lower costs and more environmentally sustainable poultry farming, which is beneficial for both producers and consumers.

## 1. Introduction

Production efficiency remains a critical focus in modern poultry farming, especially in light of rising feed costs and the growing need to minimize the environmental impact of animal production. Feed costs represent approximately 60–70% of the total expenses in poultry production [1]. As a result, genetic improvement programs aim to prioritize the selection of animals with enhanced feed utilization efficiency [2]. Residual feed intake (RFI) is a key metric for evaluating feed efficiency. RFI is generally defined as the difference between the observed feed intake (OFI) and the predicted feed intake (PFI), which is calculated based on metabolic body weight (linked to maintenance needs), egg weight and body weight gain (linked to production requirements) over a given production period [3,4]. Birds with lower-than-expected feed consumption for their production and livelihood needs have a negative RFI. Birds with a low predicted feed intake need less feed to achieve a similar body weight and production efficiency. Therefore, breeders must avoid birds with a higher feed intake through effective selection programs [5]. Feed intake is strongly influenced by changes in body weight and the weight of eggs laid. RFI can be calculated as feed intake adjusted for body weight, weight gain and the number of eggs produced during the control period (usually 4–6 weeks).

Several factors influence residual feed intake, including basal metabolic rate, activity level, plumage condition, body temperature and anatomical or morphological structure. These factors directly affect the net energy required for maintaining basic bodily functions and weight gain or egg production, which, in turn, influences the animals’ feed intake.

## 2. Materials and Methods

Publicly available databases such as Google Scholar, ScienceDirect, Scopus, PubMed and Web of Science were used to search literature sources and analyze materials. The following keywords were used for the search occurring alone or in combination: “residual feed intake”, “poultry”, “feeding efficiency”, “feeding behavior” and “environmental impact”. In addition, to broaden the scope of the search, a “snowball” technique was applied, using citation information in more recent review papers in a similar field. Articles relating to global genotypes were included; however, so were those that included studies on local poultry breeds. A total of 151 articles meeting the above criteria were found, of which 56 were used.

Peer-reviewed articles published since 2000 that were made available in full text in open access format were selected for further analysis. A few older articles with relevant information that were not referenced in the more recent literature were included. To ensure data quality, commentaries, editorials, reviews, letters, case reports and duplicate studies were excluded.

## 3. Metabolism

The rate of basal metabolism is the resultant of the previously mentioned factors. When measuring basal metabolism, it is impossible to control all the required states of energy demand. Ideally, during the measurement, the bird should not show any activity associated with additional energy demand—it should be at rest physically as well as emotionally. Usually, to determine this parameter, constant heat production is measured, which assesses basal metabolism. It is estimated that differences in metabolic rate can range from 4 to 43% between individuals of different lineages [6]. Such differences may be due to, among other things, differences in the plumage of individuals, the size of naked body parts and anatomical structure, all of which are directly reflected in the amount of energy dissipated in the form of heat, increasing the need for feed. It should also be noted that differences in energy requirements may be individual in nature, which has been confirmed by Sabri et al. [7]. However, these differences can be offset by possible use of the partial regression coefficients as selection criteria for separately improving maintenance efficiency and egg production efficiency.

## 4. Body Temperature and Arrangement of Feathers

Feathers are the main form of insulation and can therefore cause significant differences in energy metabolism. Thermal insulation depends on changes in the arrangement of feathers on the bird’s body and thus on the depth of the air layer around the body, which can affect heat loss. Studies on feed intake in well-feathered and poorly feathered hens by Gonyou & Morrison [8] and Conson [9] indirectly confirmed the relationship between plumage condition and heat loss. However, it should be noted that although the removal of feathers resulted in an increase in feed intake, the return of insulation in the form of clothing did not improve feed intake [8]. Such a result may be related to the stress caused by the procedures necessary for the experiment, or, according to the authors, it may be the result of increased effort in moving around in clothing. On a similar basis, heat loss is also affected by the area of bare body parts (crest, bells, legs): the larger they are, the greater the heat loss.

In addition, energy loss is also significantly influenced by body temperature and its anatomical and morphological structure. As for temperature, maintaining the body at a higher body temperature requires more heat production, which is negatively reflected in residual feed intake. In the case of anatomical and morphological structure, attention is primarily paid to body weight and the proportion of stored fat and protein. However, as proposed by Luiting [6], the often found negative correlation between fat content and residual feed intake indicates a presumption that maintaining body fat requires little more energy than producing it.

## 5. Behavior and Physical Activity

Another factor affecting feed intake is activity. Total heat loss during activity can be divided into activity related to muscle energy associated with work associated with movement and the physical fraction of heat loss associated with breaking the insulating layer during movement, e.g., wing flapping, shaking and feather cleaning [6]. An undeniable fact that raises the value of residual feed intake is activity related to feed intake, where the increase in RFI can be hypocritical and dictated not so much by feed intake as by feed spillage during feed intake. On the other hand, behavior such as shaking may be indicative of fearfulness or increased stress levels. Thus, a reduction in stress and fearfulness would be expected to be associated with a reduction in RFI. This was confirmed in a study by Drouilhet et al. [10] on Muscovy ducks where it was reported that lower RFI was associated with less fearfulness. It should also be noted that in studies by Morrison & Leeson [11], it was indicated that birds more efficient in terms of feed conversion had lower activity levels including shorter time spent on food intake compared to birds that were not efficient in this regard. In broilers, feeding efficiency depends on gender, among other factors, where males with high feed efficiency were characterized by fewer visits and meals per day, longer breaks between meals, less time spent at feeders and less wandering between feeding stations. In contrast, for females, the number of meals and visits per meal had no effect on feeding efficiency [12]. This result indicates a relationship between physical activity level and residual feed intake.

Physical activity is also directly influenced by bird behavior including feeding behavior. For example, in Japanese quail hens, genetic correlations have been reported between feeding traits and responses to tonic immobility [13]. This suggests that quail that use feed more efficiently and consume less feed may be less anxious and less prone to stress. The lack of a significant correlation between feeding efficiency and feeding behavior traits obtained by Drouilhet et al. [10] may indicate a limited relationship between RFI and feeding behavior. Nonetheless, others have proved that improved feed efficiency in ducks can also be influenced by certain feeding behaviors [14]. In addition, these researchers noted that ducks with high RFI prefer feeders at the edge, where they are more susceptible to environmental factors, which may indicate a link between RFI and the environment.

## 6. Calculation of RFI

To date, two primary measures of feeding efficiency have been utilized: the feed conversion ratio (FCR) and residual feed intake (RFI). The feed conversion ratio is defined as the ratio of feed intake (FI) to body weight gain in broilers or to egg weight in layers. Although RFI has been promoted by researchers as a more biologically representative measure of feed efficiency than the FCR [15], it has remained largely underutilized for the past 50 years. Both FCR and RFI-based selection lead to reduced feed intake without compromising production. However, RFI is superior to the FCR for improving feed utilization because it is independent of individual birds’ maintenance energy requirements [3]. In general, RFI is calculated as the difference between actual feed intake and expected intake, which is determined based on metabolic requirements specific to species and functional type. All formulas for calculating RFI must take into account body weight and changes in body weight, but depending on the utility, they will be adjusted for egg production for laying hens and Japanese quail [1,16] or for daily gain for meat utility [17,18,19,20,21] (Table 1).

In laying hens, attention is paid primarily not to the weight of the bird but to the number and weight of the eggs laid, as well as to egg quality traits such as protein height, yolk weight, and the percentage of protein, yolk and shell weight in the egg mass. Consequently, in egg production, the most desirable birds will be those that lay as many eggs as possible with the highest yolk percentage while using as little feed as possible. In the poultry industry, birds with the lowest possible RFI, i.e., birds that will produce the same number of eggs of the same quality, but with less feed consumption, are desirable. Compared to meat production where body weight and weight gain play a crucial role, these differences are reflected in the methods of calculating RFI. So, the general formula for calculating EFI (expected feed intake) for egg production is as follows:

EFI = aBW_i_^0.75^ + bEM_i_ + c∆BW_i_ + d,

where EFI is the expected feed intake of hen i (g); BW_i_^0.75^ is the average metabolic body weight of hen i (g0.75); EM_i_ is the egg weight of hen i (g); ΔBW_i_ is the weight gain of hen i (g); a, b and c = partial regression coefficients; d = intercept.

For meat production, the formula incorporates average daily weight gain instead of egg mass. Furthermore, the equation for expected feed intake (EFI) in meat production may vary depending on growth rate and associated traits, such as sex. Existing differences between patterns for the same species, breeds and production groups may be due to differences in the maintenance of birds, but these differences are not significant. In the case of residual feed intake (RFI), actual feed intake is measured, from which expected feed intake is subtracted (Table 1). The different RFI equations presented in Table 1 highlight the adaptability of this parameter across various poultry breeds, species and production groups. However, it is also evident that variations in bird maintenance, environmental conditions and genetic factors contribute to discrepancies in RFI calculations, necessitating further research to refine its predictive accuracy.

## 7. Heritability of RFI

RFI exhibits low to moderate heritability in broilers, chickens, turkeys and Pekin ducks [15,19,23,24,25,26,27]. Moreover, RFI plays a crucial role in enhancing poultry production and laying performance, particularly under heat stress conditions. A study on Japanese quail (*Coturnix japonica*) demonstrated that reducing RFI improved feeding efficiency and increased laying performance, especially in high-temperature environments [1]. In broilers, incorporating RFI as a selection criterion alongside growth traits such as body weight can significantly reduce production costs. Furthermore, selection based on RFI and the FCR has been effective in broilers, as both traits exhibit low to moderate heritability, with estimates ranging from 0.21 to 0.49 for RFI and 0.11 to 0.44 for the FCR [3] (Table 2).

Heritability differences in poultry species and production types arise from genetic variation, management practices and environmental conditions. Broilers show moderate heritability for RFI due to rapid growth and controlled feeding, while laying hens have slightly lower heritability because of the influence of body weight and egg production traits. These differences highlight the importance of species-specific selection strategies when implementing RFI-based breeding programs.

Feed conversion can be improved in response to selection for higher production levels and in some species by selecting for lower adult body weights [29]. A later study found that residual feed intake was a moderately heritable trait (0.47). In addition, the results obtained by Wolc et al. [30] indicate that genomic selection can be used to improve feed efficiency in laying hens, as in this study genomic prediction was higher and more durable (better maintained across generations) than prediction based on pedigree information.

Research on RFI, despite its moderate heritability, is important for improving feed efficiency in poultry production. Future studies should aim to enhance genetic evaluation models for more accurate RFI selection, considering additional factors such as gut microbiota composition, immune function and behavioral traits. As genetic selection techniques progress, RFI is expected to become increasingly significant in the sustainability and profitability of poultry production systems.

## 8. Genetic and Phenotypic Correlations

Many researchers have focused on examining genetic and phenotypic correlations between RFI and various production traits. The results vary depending on the species and production type. Genetic correlations between body weight and RFI exhibited the greatest variability, ranging from −0.87 to 0.71, for turkeys and laying hens, respectively. The lowest correlations had meat type birds. On this basis, it can be concluded that birds used for meat production have a rather negative correlation of RFI with body weight, in contrast to laying hens. These differences are due to both interspecies and functional differences, as well as differences in housing and measurement conditions. For the correlation of FI with RFI, a positive correlation ranging from low (0.21) to moderate (0.57) was observed across species. Moderate correlation strength was observed for the FCR (0.36 to 0.77). These correlations suggest that selection for negative RFI can genetically increase feed efficiency and decrease feed intake.

For phenotypic correlation, there was a low correlation of RFI with body weight for all species (−0.13 to 0.3) except turkey, where a high negative correlation was observed (−0.77). Phenotypic correlations of FI with RFI were moderate (0.4 to 0.61) for all species except Japanese quail, where high correlations were observed (0.89 and 0.91 for gray and white quail, respectively). For the FCR, a moderate correlation was observed for most species and functional types, with the exception of general utility hens and turkeys where low correlations with RFI were recorded (0.25 and 0.15, respectively). Egg production and weight had a low correlation with RFI, with a negative correlation for production and a positive correlation for weight. In turkeys, egg production was moderately negatively correlated with RFI. Therefore, it can be inferred that RFI is phenotypically independent of these traits, given the generally low phenotypic correlations between RFI and various production parameters (Table 3).

In ducks, low residual feed intake (RFI) did not show a negative effect on pectoral muscle performance but did show a positive effect on pectoral muscle shear strength. Moreover, RFI showed a positive effect on plasma triglyceride and glucose levels. These results indicate that selection for low RFI can improve feeding efficiency in ducks without affecting production efficiency. This study provides valuable insight into the biological processes underlying changes in feeding efficiency in meat ducks [17]. A study by Tixier-boichard et al. [32] found that the feed conversion ratio in laying hens was strongly negatively correlated with residual feed intake in females and less so in males. In females, total feed intake was positively linked to the number of eggs laid (0.55). Residual feed intake in females had no correlation with egg weight (−0.03), while in males (based on pedigree data), it was slightly positively correlated with egg weight (0.21). Correlations with shell thickness were low but negative, with a significant value for residual feed intake in females. In addition, eggs from hens with higher residual feed intake were characterized by thinner shells.

In addition, Yi et al. [19] showed that RFI is phenotypically independent of daily gain and average metabolic weight, but there are some genetic correlations. Selection for the feed conversion ratio (FCR) has been successfully applied to improve feed efficiency, but the use of the “ratio” trait has mathematical limitations because selection pressure is usually exerted on the component traits of the FCR in a non-linear manner. Moreover, RFI has little or no correlation with production traits, indicating that genetic improvement of RFI within a selection index can be made without FCR-related interference [35].

In a study by Wolc et al. [30], it was proven that hens that consume more feed and have higher residual feed intake (lower productivity) genetically tend to lay slightly more eggs with higher yolk weight and protein height. In contrast, shank length, tailbone length and rectal and comb temperature showed higher values in birds with high RFI, suggesting increased heat production or dissipation. The opposite results were obtained by Galal & Mahorus [36] where the commercial hen line had both better production results and a better RFI score; however, the local breed had better shell quality and better resistance after PHA-P (Phytohemagglutinin-P) injection. In addition, Bordas et al. [37] found a significant correlation between feed conversion and residual feed intake in Rhode Island Red hens: birds with low residual feed intake had improved feed efficiency. In contrast, no significant negative changes in egg production and egg quality traits were observed for Japanese quail [14]. For ducks, lower RFI values increased the number of eggs produced annually, as well as hatchability and fertility rates [28]. In addition, Pakdel et al. [38] noted a weak positive genetic correlation between feeding efficiency and ascite-related traits in broilers. This indicates that more efficient birds are slightly more susceptible to ascites. Non-significant differences may affect egg production, egg quality traits and carcass traits to a greater or lesser extent depending on the species and type of birds. This suggests that RFI can be used as a selection criterion to improve feed efficiency without significant negative differences in the aforementioned parameters. On the other hand, Wood & Willems [34] noticed negative genetic correlation between RFI and fertility and hatch of fertile (−0.29 and −0.44, respectively).

## 9. Genomic Basis

The genomic basis of RFI variation remains to be determined. Selected analyses have shown that many hundreds of genes are associated with variation in RFI values [39]. However, RFI is not a performance parameter and should not be used as the sole selection criterion. The ability of the current method to estimate performance values for metabolic body weight and weight gain provides geneticists with additional parameters to use to distinguish between animals with similar RFIs [15]. Based on analysis of actual egg production data and quality traits in laying hens, the study by Wolc et al. [40] shows that the accuracy of estimated breeding values (EBVs) based on dense marker data is on average higher than that based on pedigree data. Improving the accuracy of all methods can be achieved by increasing the size of the training data set. It should be noted, however, that this improvement has not occurred to the extent predicted by the literature, which may be the result of population selection.

In a study of a local breed of hens by Izadnia et al. [41], QTL analysis showed that 63 genes were linked to feed conversion traits. In addition, the researchers identified some important candidate genes for further improvement programs in Iran’s Isfahan native breed, including the *RSAD2*, *IL15*, *LIPI*, *EGR1* and *DUSP16* genes. Ye et al. [42], using genome-wide association analysis (GWAS) in their study, identified seven SNPs (single-nucleotide polymorphisms) and five candidate genes associated with RFI, twenty SNPs and one candidate gene (inositol polyphosphate multikinase) associated with average daily gain, and one SNP and one candidate gene (alpha subunit of coat protein complex) associated with average daily feed intake. After transcriptomic analysis between the high- and low-RFI groups, 38 trait-enhancing genes and 26 trait-lowering genes were identified in the high-RFI group. A study by Xu et al. [43] found a region of the genome that was identified as a key candidate region affecting the energy use efficiency of chickens, as it contained genes related to lipogenesis, social behavior and immunity.

In the study by Liu et al. [44], in addition to finding candidate genes for the local breed as well as for broiler chickens, the genes were shown to be associated with other functions. Thus, annotation of the functions of RFI-related genes showed that Beijing-You (local breed) genes were enriched in lipid and carbohydrate metabolism, as well as the phosphatase and tensin homolog (PTEN) signaling pathway. In broilers, RFI-related genes were enriched in the feeding behavior process and the cAMP-responsive element binding protein (CREB) signaling pathway. Both breeds showed enrichment in physiological processes related to body development.

Additionally, genomic selection has emerged as a promising tool for improving RFI heritability estimates. Research indicates that genomic prediction provides higher accuracy and stability across generations compared to traditional pedigree-based selection methods. This advancement suggests that genetic markers associated with RFI can be effectively utilized to enhance selection efficiency in poultry breeding programs.

## 10. Physiological Basis

Bottje & Carstens [45] found that individuals with low RFI showed better coupling in the electron transport chain in muscle mitochondria, which were provided with an energy substrate bound to NADH (nicotinamide adenine dinucleotide), compared to individuals with high RFI. However, there were no differences in coupling in the electron transport chain in pectoral muscles or leg muscles between broilers with high and low feed efficiency when mitochondria were provided with succinate as an energy substrate. These results illustrate the need for further research to investigate the mechanisms responsible for inter-individual variation at the mitochondrial level. In a study by Clark et al. [46], it was observed that high-feed-efficiency (HFE) hens consumed less feed and chose a diet with a higher crude ash content and lower gross energy compared to LFE (low feed efficiency) hens. LFE hens also spent more time eating and walking, but spent less time resting, drinking, cleaning themselves and pecking their cages compared to HFE hens. In addition, selection for lower RFI improves feed efficiency without affecting growth performance, slaughter rate or meat quality of ducks. The results of this study also indicate that reduced mitochondrial energy metabolism may contribute to the RFI of slow-growing ducks, with the *PRKAG3* gene playing a key role in this biological process [47].

Bernard et al. [48] proved that a richer and more diverse microbiota can play a role in increasing feed efficiency, but nevertheless in a diet-dependent manner. In addition, the observed taxonomic differences in the composition of the gut microbiota may correlate with changes in starch and fiber digestion, as well as in the production of short-chain fatty acids. Consequently, this means possible optimization of nutrient absorption by hens more efficient in terms of RFI through the activity of fibrolytic bacteria. The effect of microbiota on RFI is dependent on the part of the gastrointestinal tract. The cecal microbiota accounts for the largest percentage of variance compared to the microbiota of the duodenum, jejunum, ileum and feces. Additionally, higher abundance of *Lactobacillus*, *Corynebacterium*, *Coprobacillus* and *Slackia* and lower abundance of *Akkermansia muciniphila* and *Parabacteroides* in the cecum are associated with better feed efficiency [49]. Triglyceride metabolism also plays an important role in RFI levels. Thus, ducks with lower RFI were characterized by significantly higher expression levels of *PPARγ*, *GK2* and *LIPE* (peroxisome proliferator activated receptor γ, glycerol kinase 2, lipase E) genes, which had a significant negative correlation with the FCR and RFI [50]. This suggests that the expression of genes correlated with metabolism and transport is upregulated in the duodenum of birds with high feed efficiency. Additionally, these genes are important genes affecting RFI and may serve to further study the mechanism of RFI at the cellular and molecular levels.

In laying hens, genetic differences in the ability to metabolize gross feed energy were found to be limited; the coefficient of variation was 1–3%. The main component of variation in RFI appears to be variation in metabolic energy: differences in physical activity, plumage density, basal metabolism, bare skin area (comb, legs) and body temperature [6]. In a study by Gabarrou et al. [51], it was proven that hens selected for high residual feed intake (RFI+), despite spending the same time on feed intake as hens with low residual feed intake (RFI-), consumed more frequent but shorter meals. In addition, RFI+ hens ate significantly more feed than RFI- hens. No significant changes in plasma concentrations of triglycerides, phospholipids, uric acid, glucose or insulin were observed between the lines, suggesting the use of similar thermogenic pathways regardless of the direction of selection.

## 11. Impact on Environmental Emissions

At the moment, literature data do not provide much information on the environmental impact of selection for high feed efficiency. The largest carbon footprint in poultry production is recorded for meat production; therefore, selection for feed efficiency is particularly important in broiler production [41]. Studies on broilers indicate that selection toward lower feed conversion rates reduces fresh excreta by 41.3% [34]. In addition to this, these indices have a high genetic correlation with fresh excreta, which offers the possibility of improving the genetic amount of excreta through genetic improvement of feeding efficiency [52]. Moreover, a reduction in N_2_O and CH_4_ levels may occur due to a reduction in enteric fermentation, along with improved feed conversion [53]. In addition, data for other species such as cattle and pigs describe the effect of selection for increased nutritional efficiency on the environment as positive or neutral [54,55,56]. This makes this aspect interesting and important for future research.

## 12. Suggestions for Future Research

Despite the advancement of knowledge in the field of selectively breeding birds for better feed utilization, there are still many questions that need to be answered but new solutions are emerging that can bring significant results in improving residual feed intake in poultry farming. Therefore, the authors expect to address the following questions in future research.

1. The use of artificial intelligence (AI) and computer vision techniques to quantitatively identify and model feeding behavior to improve analytical methods for RFI estimation and prediction of expected results taking into account the species, functional type and structure of the breeding flock.
2. Given that the measurement of RFI is time-consuming and labor-intensive, it seems reasonable to look for traits highly correlated with RFI through, for example, analysis of feeding behavior with the possibility of using image analysis methods via AI.
3. In the current literature, there is no consensus on the direction of correlations between RFI and other traits or they are isolated analyses (e.g., correlations with reproductive traits), which requires future research.
4. Given the impact of poultry production on environmental pollution levels, it is important to examine to what extent improvements in RFI can contribute to lower greenhouse gas emissions for both meat and layer production.
5. Given the increasingly lower costs of molecular analysis and gene editing, these techniques are becoming increasingly available in poultry breeding, which calls for a broader analysis of their use in improving RFI in poultry.

## 13. Conclusions

In conclusion, reducing residual feed intake (RFI) in poultry can significantly lower production costs and enhance the overall efficiency of poultry production. Precise calculation of RFI is essential for accurate selection of individuals, but it is essential to consider the demands of a particular species, functional type and the individual characteristics of birds for an accurate assessment. Research on RFI demonstrates substantial potential for improving laying performance, egg quality and the effective utilization of feed components. The heritability of RFI suggests that selective breeding can improve feed efficiency over generations. Additionally, genetic and phenotypic correlations indicate trade-offs between RFI and other production traits, requiring a balanced approach in breeding programs. Moreover, selective breeding can identify individuals with superior nutrient utilization capabilities, helping to minimize resource losses. Advances in genomic research provide insights into the genetic basis of RFI, enabling marker-assisted selection for improved efficiency. Furthermore, physiological studies highlight mechanisms such as gut microbiota composition and nutrient absorption efficiency that contribute to RFI variation. However, it is important to note that selection for reduced RFI may have limitations, particularly in terms of the energy reserves required to cope with environmental stress. But by integrating the aforementioned factors, breeders can optimize feed efficiency while maintaining overall production levels and welfare.

## Figures and Tables

**Table 1:** Computed residual feed intakes (RFIs) of different populations of poultry.

Breed/Species	Production Type	Equation	Reference
Chicken	Egg production	RFI = FI − b_0_ + b_1_ × MBW^0.75^ + b_2_ × EM + b_3_ × BWG	[22]
Chicken	Broiler	RFI = FI − (b_0_ + b_1_ MMBW + b_2_ BWG)	[18]
Chinese local chicken (male)	Growing	EFI = 0.381 MBW^0.75^ + 1.09 ADG − 5.14	[19]
Chinese local chicken (female)	Growing	EFI = 0.069 MBW^0.75^ + 1.48 ADG − 37.8	[19]
Chinese local chicken	Growing	RFI = ADFI − (b_0_ + b_1_ × MMBW + b_2_ × ADG)	[19]
Japanese quail	Egg production	EFI = aBW_i_^0.75^ + bEM_i_ + c∆BW_i_ + d	[1]
Japanese quail	Egg production	RFI = FI − (b_1_ × MBW) − (b_2_ × WG)	[16]
Duck	Meat production	RFI = FI − (a + b_1_ × MBW^0.75^ + b_2_ × ADG)	[17]
Duck	Meat production	RFI = FI − (b_0_ + b_1_ BW^0.75^ + b_2_ WG)	[20]
Turkey	Meat production	RFI = FI − (b_0_ + b_1_ MMW + b_2_ WG)	[21]

EFI—expected feed intake; MBW^0.75^—metabolic body weight; MMBW—mid-test metabolic body weight; BW_i_^0.75^—mean metabolic body weight of hen i (g0.75); EM_i_—egg mass production of hen i (g); ∆BW_i_,—body weight gain (g); a, b, b_1_, b_2_ and c—partial regression coefficients; d and b_0_—intercept; WG—weight gain; ADG—average daily gain; ADFI—average daily feed intake; MMW—metabolic mid-weight.

**Table 2:** Sample heritability for some poultry species.

Species/Line/Breed	Production Type	Heritability	References
Chicken	Meat production	0.35	[15]
Chicken	Broiler	0.38	[18]
Chicken	Broiler	0.29	[24]
Chicken (Keniyan local breed)	Growing period	0.43	[23]
Chicken (Keniyan local breed)	Laying period	0.30	[23]
Chicken (Tianlu Black chicken)		0.28	[19]
Turkey	Meat production	0.23	[25]
Pekin duck	Meat production	0.41	[26]
Muscovy duck	Meat production	0.83	[28]
Duck (INRA I444 strain)	Laying period	0.24	[27]

**Table 3:** Genetic and phenotypic correlations between RFI and some productive traits.

Trait	Genetic Correlation	Phenotypic Correlation	Production Type	Species	References
BW		−0.003	Laying	Gray quail	[1]
		0.002	Laying	White quail	[1]
	−0.52	−0.04	General	Chicken	[23]
		−0.13	Meat	Chicken	[31]
	0.71	0.004	Laying	Chicken	[32]
	0.27	0.3	Broiler	Chicken	[18]
		0.004	Meat	Duck	[33]
	0.12		Laying	Duck	[27]
	−0.87	−0.77	Meat	Turkey	[25]
FI		0.89	Laying	Gray quail	[1]
		0.91	Laying	White quail	[1]
	0.57		Laying	Chicken	[32]
	0.48	0.4	Broiler	Chicken	[18]
		0.49	Meat	Duck	[33]
	0.21	0.61	Meat	Turkey	[25]
FCR		0.55	Laying	Gray quail	[1]
		0.49	Laying	White quail	[1]
	0.41	0.25	General	Chicken	[23]
		0.73	Meat	Chicken	[31]
	0.69	0.65	Meat	Chicken	[19]
	0.77	0.79	Broiler	Chicken	[18]
	0.36	0.15	Meat	Turkey	[25]
Egg production %		−0.07	Laying	Gray quail	[1]
		−0.05	Laying	White quail	[1]
	−0.34		Meat	Turkey	[34]
Egg weight		0.11	Laying	Gray quail	[1]
		0.18	Laying	White quail	[1]
	−0.03		Laying	Chicken (female)	[32]

BW—body weight; FI—feed intake; FCR—feed conversion ratio.

## Data Availability

The raw data supporting the conclusions of this article will be made available by the authors upon request.
